# Supplementary material for: Prospective cohort study of unselected nulliparous women with a nested randomised controlled trial of screening using the sFLT1:PlGF ratio, ultrasound and maternal characteristics and intervention using enhanced monitoring and early delivery: study protocol for the POPS2 cohort and randomised controlled trial
Source: BMJ Open. 2026 Aug 7;16(8):e122043. doi: 10.1136/bmjopen-2026-122043 (PMC13475102; doi:10.1136/bmjopen-2026-122043)
Supplement: Supplementary file 1 [file bmjopen-2026-122043.reviewer_comments.pdf]

PEER REVIEW HISTORY

BMJ Open publishes all reviews undertaken for accepted manuscripts. Reviewers are asked to complete a checklist review form and are provided with free text boxes to elaborate on their assessment. These free text comments are reproduced below.

ARTICLE DETAILS

Title (Provisional)

A prospective cohort study of unselected nulliparous women with a nested randomized controlled trial of screening using the sFLT1:PlGF ratio, ultrasound and maternal characteristics and intervention using enhanced monitoring and early delivery: study protocol for the POPS2 cohort and randomized controlled trial

Authors

Smith, Gordon C.S.; Sutton-Cole, Amy; Dyer, Ellen; Sovio, Ulla; Cook, Emma; White, Ian R; Charnock-Jones, Stephen

---

This article was not externally reviewed at BMJ Open. Protocols that have been independently assessed prior to submission to BMJ Open are usually fast-tracked to publication on the grounds that further substantial changes will not be possible. This independent assessment will usually be external, independent review for both a substantial grant award from a non-commercial or government funder and ethics approval.
