# Supplementary material for: Prospective cohort study of unselected nulliparous women with a nested randomised controlled trial of screening using the sFLT1:PlGF ratio, ultrasound and maternal characteristics and intervention using enhanced monitoring and early delivery: study protocol for the POPS2 cohort and randomised controlled trial
Source: BMJ Open. 2026 Aug 7;16(8):e122043. doi: 10.1136/bmjopen-2026-122043 (PMC13475102; doi:10.1136/bmjopen-2026-122043)
Supplement: Supplementary file 2 [file bmjopen-16-8-s001.docx]

**Place ID**

**sticker here**

**POPS2 CONSENT FORM**

Title of Project: Pregnancy Outcome Prediction Study 2

Name of Researcher: Prof. Gordon C S Smith

**Please initial box:**

1.	I confirm that I have read and understand the "Pregnancy Outcome Prediction Study 2 (POPS2) participant information leaflet” Version ______ , date ___/___/___ " and have had the opportunity to ask questions.

2.	I understand that my participation is voluntary and that I am free to withdraw at any time, without giving any reason, without my medical care or legal rights being affected.

3.	I understand that the relevant sections of my medical record and my baby’s medical record will be accessed by the research team for the purposes of the study and I give my permission for this access.

4.	I understand that my samples and my baby’s samples will be analysed, including DNA, and I give my permission for this analysis. I understand that I will not be informed of the results.

5.	I understand that the information collected about me will be used to support other research in the future, and may be shared anonymously with other academic and commercial researchers external to the project within the UK and beyond

6.	I understand that my subsequent health records and my baby’s subsequent health and educational records may be used to study long term outcomes

7.	I agree to my GP being informed of my participation (optional, please put a cross in the box if you do not wish your GP to be informed)

8.	I agree to take part in the above study.

**Name of patient**	Date	Signature

**Name of person taking consent**	Date	Signature
